# Meta-analysis of the impact of plant invasions on soil microbial communities

**DOI:** 10.1186/s12862-021-01899-2

**Published:** 2021-09-08

**Authors:** Nardi Torres, Ileana Herrera, Laurie Fajardo, Ramiro O. Bustamante

**Affiliations:** 1 Centro de Ecología, Instituto Venezolano de Investigaciones Científicas (IVIC), A.P. 20632, Caracas, 1020-A Venezuela; 2 Universidad Espíritu Santo, Escuela de Ciencias Ambientales, Samborondón, 091650 Ecuador; 3 Sección Botánica, Instituto Nacional de Biodiversidad (INABIO), 170501 Quito, Ecuador; 4 Departamento de Ciencias Ecológicas, Instituto de Ecología y Biodiversidad, Facultad de Ciencias, Universidad de Chile, Las Palmeras No 3425, Santiago, Chile

**Keywords:** Soil microbial communities, Biodiversity of bacteria, Categorical model, Biological invasions

## Abstract

**Background:**

One of the ecological impacts of exotic plant invasions may be alteration of the soil microbial community, which may cause changes to the diversity, richness and function of these communities. In order to explore to what extent invasive plants affect the soil microbial community, we performed a meta-analysis based on 46 scientific articles to document the effect of invasive plants on species richness and diversity of bacteria and fungi. We conducted our study across a range of invaded ecosystems including native communities, and evaluated biomass, richness and diversity. We use a random effects model to determine the increase or decrease in the values of the response variables in the presence of invasive plants.

**Results:**

The results indicated that the response variable that changed with the invasion of plants was the diversity of bacteria. Bacterial diversity in the soil increases with the presence of invasive plants, specifically herbaceous plants producing allelopathic substances growing in forest ecosystems of temperate zones.

**Conclusions:**

We provide evidence that invasive plants affect the soil biota differentially; however, it is important to consider more variables such as the N and C cycles, since these processes are mediated by soil biota and litter, and chemical compounds released by plants influence them. Changes in bacterial diversity have consequences for the nutrient cycle, enzymatic activity, mineralization rates and soil carbon and nitrogen content.

**Supplementary Information:**

The online version contains supplementary material available at 10.1186/s12862-021-01899-2.

## Background

A plant becomes invasive when naturalized populations are able to spread across a new range beyond introduction sites [19]. One of the ecological impacts generated by invasive plants is the alteration of the soil microbial community (SMC), which entails changes in the availability of nutrients [24, 47, 52] and a reduction in biodiversity [41, 42]. Specifically, invasive plants affect free-living fungi (FLF), bacteria, and arbuscular mycorrhizal fungi (AMF), which are critical for nutrient fluxes, resource availability [46] and the composition and structure of native plant communities [7, 28].

One well-known mechanism to explain how exotic plants can affect soil microbiota is through allelochemicals (“the novel weapon hypothesis”). Basically, these plants exude chemical substances which besides reducing survival and regeneration of native plants [18, 47], can significantly change the SMC in the rhizosphere [43, 49], affecting decomposition processes [3], metabolizing labile and recalcitrant substrates [9] such as nitrogen mineralization and nitrification [10], and modifying soil enzyme activities [22], as well as changes in the SMC as a result of other ecosystem impacts [11].

In summary, we have some idea about the impact of invasive plants on soil microbes [17, 48, 51]; however, we are far from possessing an integrated body of knowledge to make generalizations about the interactions between invasive plants and soil microbes [1, 20, 51]. One way to address this question in a general way is to conduct a meta-analysis. This procedure examines the results of multiple studies in order to detect general patterns [15]. In the present study, we conducted a meta-analysis to examine the effect of invasive plants (those that produce allelopathic substances vs plants that do not), on the SMC.

In particular, we estimated the effects of plants that produce allelopathic substances on the SMC. Following the “novel weapon hypothesis,” we expected negative effects on the SMC from plants producing allelopathic compounds compared to plants that do not produce them.

## Results

### Case description

We found 211 cases published in 46 scientific papers (studies) that evaluated the impact of 50 species of invasive plants (belonging to 15 families) on the SMC (Additional file 1). Eighty-three percent of cases were in temperate zones, and 17% in tropical and sub-tropical regions. We found only one case study for the Neotropics. Forty-seven percent of cases were conducted in forest ecosystems. Six percent of cases were conducted in wetlands and disturbed areas. Interestingly, 39% of cases were conducted in controlled conditions (laboratories and greenhouses).

Six species were the most studied for being very aggressive, which corresponded to 35% of cases (N = 76). These species are *Alliaria petiolata* (Brassicaceae; 16 cases); *Berberis thunbergii* (Berberidaceae; 11 cases); *Ageratina adenophora* (Asteraceae; 14 cases); *Impatiens glandulifera* (Balsaminaceae; 13 cases); *Jatropha curcas* (Euphorbiaceae; 12 cases) and *Bromus tectorum* (Poaceae; 10 cases); (Additional file 1).

**Fig. 1:**
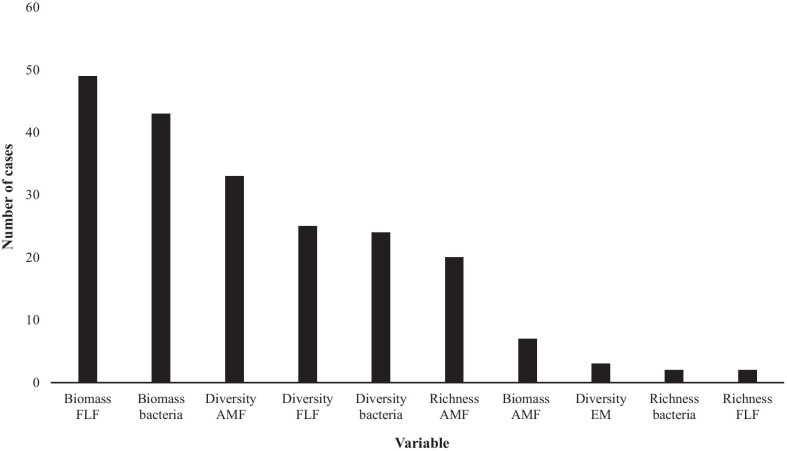
Number of cases per response variable evaluated (N = 204). FLF: free-living fungi, AMF: arbuscular mycorrhizal fungi, EM: ectomycorrhizal

We found that 29% of the cases included plants producing allelopathic substances (13 species; 62 cases), while 71% of cases (37 species; 149 cases) did not produce such substances. The FLF biomass (49 cases) and bacterial biomass (43 cases) were the response variables most used to examine the effects of invasive plants on the SMC (Fig. 1).

We used only 201 cases for the heterogeneity test, because some variables such as ectomycorrhizal diversity (3 cases), bacteria richness (2 cases), bacteria density (3 cases), and richness of FLF (2 cases) did not have enough studies for this test.

### Effect size on response variables

Considering all cases, there was a significant variability in the effect sizes of all invasive plants over all response variables evaluated (*Q*_*t*_ = 426.50; d.f. = 209; P < 0.0001). Mean effect sizes between response variables examined did not differ significantly among cases (Q_*b*_ = 12.30; d.f. = 9; P = 0.19676) in magnitude or direction. The mean effect size within response variables (Q_*w*_ = 414.20; d.f. = 200; P < 0.0001) was significant.

**Fig. 2:**
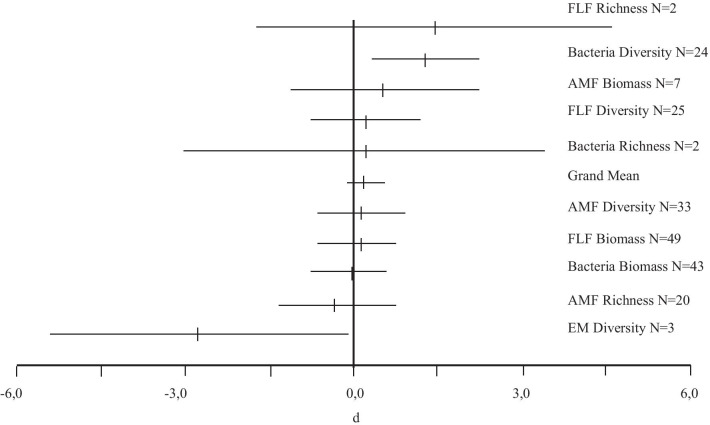
Effect size (*d* of Hedges) of invasive plants on different response variables of the SMC. SMC: soil microorganisms communities; FLF: free-living fungi; AMF: arbuscular mycorrhizal fungi

For 9 of the 10 response variables examined, the confidence interval (CI) of the mean effect size included zero, and the effect size was not significant (Fig. 2). Therefore, for these response variables, we could not support the hypothesis that the invasive plants affect these variables. However, we detected significant and positive effects of invasive plants on bacterial diversity ($E_{j}^{ + }$ = 1.25; IC_95%_ = 0.28–2.21; *n* = 24) (Fig. 2).

**Fig. 3:**
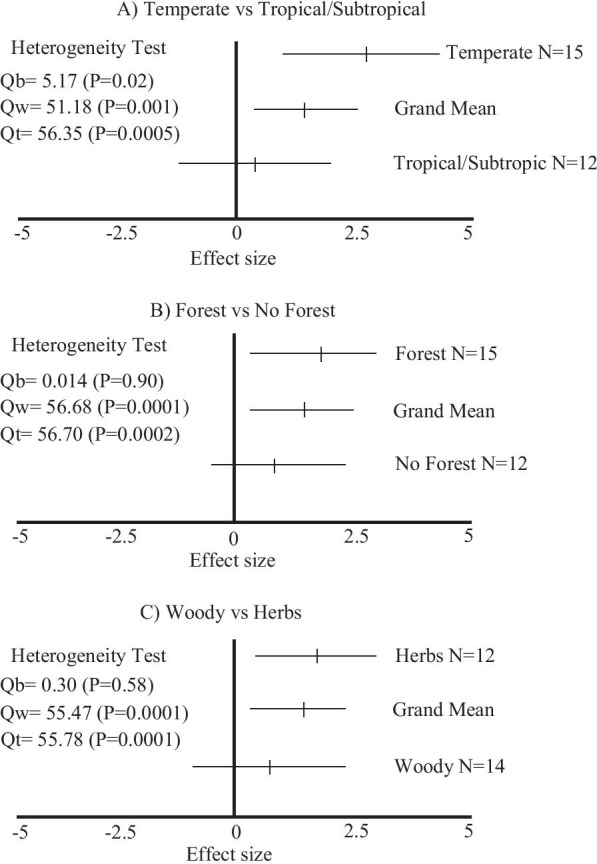
Heterogeneity test of effects of allelopathic substances on bacterial diversity in three conditions: **a** temperate vs tropical/subtropical region, **b** forest and non forest ecosystems, **c** woody and herbaceous invasive plants. *Q*_*b*_ variance of a response variable between different studies. *Q*_*w*_ variability within groups. *Q*_*t*_ variance of the cumulative effect size between different cases

The random effects model revealed that bacterial diversity was affected positively for allelopathic substances from invasive plants in three paired comparisons (*Q*_*t*_ values, Table 1). In summary, the effect of allelopathic substances produced by invasive herbs growing in temperate zones forest ecosystems positively affected bacterial diversity, as can be observed in Fig. 3.

**Table 1:** Effect of allelopathic substances on the diversity and biomass of AMF, bacteria and FLF in three categories

Response variables		Temperate vs. tropical-subtropical	Forest vs. non-forest	Woody vs. herbaceous
AMF	Diversity	*Q*_*b*_ = 0.02 P = 0.91	*Q*_*b*_ = 4.92 P = 0.11	*Q*_*b*_ = 0.90 P = 0.49
Bacteria	Biomass	*Q*_*b*_ = 0.09 P = 0.78	*–*	*Q*_*b*_ = 0.03 P = 0.87
	Diversity	*Q*_*b*_ = 4.12 P = 0.12	*Q*_*b*_ = 1.24 P = 0.40	*Q*_*b*_ = 0.97 P = 0.46
		***Q***_***t***_** = 27.06** **P = 0.02**	***Q***_***t***_** = 28.19** **P = 0.02**	***Q***_***t***_** = 32.87** **P = 0.0004**
FLF	Biomass	*Q*_*b*_ = 4.08 P = 0.13	*–*	*Q*_*b*_ = 0.04 P = 0.90
	Diversity	*Q*_*b*_ = 2.88 P = 0.23	*Q*_*b*_ = 4.92 P = 0.11	*Q*_*b*_ = 0.60 P = 0.58

*Q*_*b*_ describes the variation in effect sizes that can be ascribed to differences between categories. *Q*_*t*_ assesses whether the effect sizes are homogeneous. Bold letters indicate significant values

(–) These analysis were not carried out because some of the studies that evaluated bacterial biomass and FLF biomass were carried out under greenhouse conditions

The normal quantile showed that the effect sizes are normally distributed (Additional file 2).

### Publication bias

We found no correlation between the number of cases and effect sizes (Spearman test, *r* = −0.133; *p* = 0.189) which suggests no publication bias in the total cases evaluated. This result is also confirmed by the funnel-shaped distribution of the data points (Additional file 2), which, according to Palmer [34], would be expected in the absence of a sampling bias.

## Discussion

The meta-analysis showed that alellophatic substances produced by invasive plants had only significant and positive effects on bacterial diversity. Some of the studies were carried out in greenhouses simulating a comparison of the SMC between invaded and non-invaded areas. One limitation of the recorded studies is the duration of the experiments (< 6 months), thus probably not giving enough time to detect microbial responses to invasive plants [44, 47]. For future research, it would be advisable to integrate greenhouse and field experiments, which can complement the information obtained for invasive plants, thus giving us a more realistic picture to understand invasive plant and soil biota interactions.

Our study also reveals a geographic research bias; most of the data analyzed comes from studies on herbaceous plants (104 cases) from temperate areas of North America and Europe, while there were fewer studies in tropical areas. This bias seems to be a general pattern in invasion ecology studies [35, 36]. We also found that studies of the impact of invasive plants on soil microorganisms have concentrated on six species (*Alliaria petiolata, Berberis thunbergii, Ageratina adenophora, Impatiens glandulifera, Bromus tectorum, Jatropha curcas*), these being 35% of cases (76 studies).

Some invasive plant species (24 cases; 9 species) exert significant impact on bacterial diversity. Several studies have suggested that invasive plant species may modify the functioning of ecosystems by altering SMC (e.g., [14, 22]. A comprehensive literature review published by Pysek et al. [35] reveals that invasions by exotic plants tend to increase the richness and abundance of soil biota. Compared to native species, invasive plant species generally produce more leaf litter (49%) that is of better quality (lower C:N ratio) [27]. The greater quantity and quality of litter increases the C available in the soil, a source of energy for the SMC, which could allow the establishment of a more diverse and abundant SMC [52]. Our results partially corroborate these results, our categorical model revealed that invasive plant species have a significant effect on the diversity of bacteria in the soil, while they do not generate significant effects on the other components of the SMC [17, 47].

A recent meta-analysis comparing the effects of invasive species on the SMC from litter and the rhizosphere reports that litter increases the biomass of soil bacteria due to nutrient intake, while changes generated by the rhizosphere during the invasion decrease the biomass of bacteria [52]. The authors attribute this result to the fact that litter accumulation can have positive effects on bacterial communities [11, 12], while radical exudates (organic acids, allelopathic substances and hormones) could inhibit bacterial biomass. On the other hand, the meta-analysis performed by Meissner et al. [30] reports null effects of allelopathic substances on the biomass of bacteria in the soil. Our results are somewhat consistent with these findings, we also found that allelopathic substances released by invasive plants have no effect on bacterial biomass [22, 24], about 29.4% of invasive plant species in our database were reported to have allelopathic effects (Additional file 1), which may partially explain the absence of suppressive effects from the roots of invasive plants on bacterial biomass. Specifically, Meissner et al. [30] found that neither the litter nor the exudates from the roots of the invasive plants have effects on FLF biomass. This result (and ours) may be attributable to the fact that the effect size values for the different categories were quite variable, indicating that the FLF biomass change is contingent on the kind of invasive species as well as the ecosystem type.

Little has been done to investigate how AMF communities can be affected by invasive plants. Our meta-analysis suggests an absence of effects caused by invasive plants on the AMF community; however, certain specific studies indicate significant effects. For example, Vogelsang and Bever [49] found evidence of a reduction in mycorrhizal fungi density by nonnative plants. More recently, Rezácová et al. [38] found that invasions by five nonnative plant species altered composition of the AMF community and reduced the diversity of AM fungi in the soil and in the roots of some native plant species. However, neither of the two studies could be included in this meta-analysis because they did not meet the selection criteria established in our study [38, 50]. The results obtained in our study can be explained because the invasive plants are associated with a wide range of AMF species widely distributed in regions where they are introduced [31, 33, 39]. This may be favorable to inducing the naturalization and expansion process [32, 37] and explain why invasive plants do not alter this community.

A result of different evolutionary trajectories of invasive plants is the impressive number of different biochemicals produced by plants [6], over 100,000 different low-molecular-mass natural products have been identified in plants [4, 13]. Unexpectedly, we found that bacterial diversity was positively affected by allelopathic substances produced by invasive herbs in temperate regions; this result occurs because the SMC have an adaptation restricted to a few chemical compounds in these regions. When exudates or secondary metabolites from invasive plants enter the soil, bacteria feed on them and increase their diversity, because in the absence of these, they are not able to use organic matter as a source of energy [45]. The diversity and concentration of secondary metabolites appears to be greater in the tropics than in temperate ecosystems; in fact, its incidence in tropical flora doubles the flora of temperate zones and declines with elevation [29]. In contrast, in tropical areas, soil microorganisms have been adapted to a wide variety of substances over time, and are able to tolerate a wide variety of exudates, thus maintaining the diversity and abundance of organisms. Closely related plants and soil microorganisms may differ in their sensitivity to the same biochemical and allelochemical substances when they are from different continents, while distantly related species may have converged to similar sensitivities if they are from the same region. This suggests that plants and soil microorganisms can evolve tolerance to the unique rhizosphere biochemistry of co-occurring species with independent phylogenetic histories [6].

Physiological traits that contribute to the establishment and expansion of invasive plants can have an impact on ecosystem processes. Allison and Vitousek [2] evaluated initial leaf litter properties, decomposition rates, and nutrient dynamics in 11 forest plant species of the Hawaiian Islands. They found a 50-fold variation in litter decomposition rates, decomposition in native plants decreased (0.2–2.3 yr^−1^) and that of invasive plants increased (1.4–9.3 yr^−1^) in the forest. In another study conducted in a Long Island forest, New York, USA, Ashton et al. [3] evaluated the differences in decomposition of the litter of native and exotic plants in mesic hardwood forests. They found that litter decomposition and released nitrogen of alien species were significantly faster than in litter from native species, and the litter from all species types decomposes substantially faster at invaded sites in the forest. The greatest decomposition of the leaves of invasive plants in forest is associated with high specific leaf areas, rapid growth rates, and high leaf nutrient concentrations, which improve leaf litter quality and increase decomposition rates and nutrient cycling [2]. These results suggest that invasion by exotic plant species in forests alters the decomposition and nutrient cycle of soil, regardless of differences in litter quality specific to native and exotic species [3]. The addition of new resources that come from invasive plants brings benefits for bacterial diversity. The contribution of these resources could promote short-term changes in the microbial community of the soil [23] and bacterial reproduction [26]. The decomposition rate of organic matter in the forest floor is higher than in other ecosystems because there is more moisture and greater presence of disintegrating fauna that will fractionate the material, since vegetation is denser and there is greater microbial potential, which will be the main factor responsible for mineralization [21].

The characteristics of invasive plants and the taxonomic group they belong to have a significant impact on the diversity of the microbial community [35]. The plant life form had a positive and significant effect on the diversity of bacteria. A possible explanation for these results is that bacteria recognize the substances produced by invasive herbs as resources, thus enabling an increase of their diversity, further studies are required to test this hypothesis.

## Conclusions

Bacterial diversity was the unique microbial variable that was affected by alellopathic substances released by herbaceous invasive plants in temperate forests. A possible explanation for this result is that in the temperate forests, these plants release a smaller variety of secondary metabolites, thus enabling bacteria species to use them as resources. This unexpected result could be considered a significant contribution to invasion ecology. However, further field and greenhouse studies are required to exhaustively evaluate the role of exotic plants on soil bacterial communities.

Future studies should also consider a more mechanistic approach, including the nutrient cycles in which soil microorganisms are involved, as well as life traits of the plants, dependence on AMF, land use history and competition between native and exotic plants.

## Methods

We used ISI Web Knowledge (http://apps.isiknowledge.com) and Google Scholar (http://scholar.google.es) to search for study cases. We selected the following keywords: (i) invasive plants and exotic plants AND mycorrhizal, (ii) bacterial and fungal communities AND impact of invasive plants. The response variables were biomass, richness and species diversity of free-living fungi (FLF), bacteria and arbuscular mycorrhizal fungi (AMF). For the ectomycorrhizal (EM), we considered only diversity because it was the only variable studied in the articles.

We must clarify the difference between cases and studies. Studies refers to selected articles that meet our criteria. When a response variable is evaluated under the effect of different invasive plant species, different seasons or years, different invasion gradients, or different areas in the same ecosystem, we consider these studies as independent, because they represent different examples of ecological impacts and we call them cases. Thus, a study can have one or more cases. Although this could result in pseudo-replication, this approach has been accepted due to the small number of articles published on this topic (e.g., Villa et al. [47]).

When a response variable was measured at different times; for instance, SMC composition in different seasons [5, 25], the values of the last season were considered for the meta-analysis. If the response variable was measured in different sampling years, we took the mean value of each year as independent (for example, Dieng et al. [8]). When an invasive plant species was studied along a gradient or at different densities (high, medium, low), only conditions with high and low abundance were considered as cases. In some cases, we examined the effect of invasive plants on SMC, considering different localities but within similar ecosystem types. In other studies, where data were presented in time series, we selected the last values and in those studies that included a transition between invaded and non-invaded zones, we selected the zones with the highest abundance of the invasive plant.

The following criteria were used to select the investigations to evaluate the effect of invasive plants on soil microorganism communities (SMCs): (i) studies that included invaded and non-invaded areas in natural and semi-natural ecosystems; (ii) studies that evaluated the inherited effect of the invasive plant; (iii) studies that evaluated the effect of invasive plants on SMC in the field and greenhouse and (iv) studies that considered the effect of one or more invasive plant species on SMC.

The following studies were excluded: (i) those carried out in agricultural systems; (ii) those which evaluated negative and positive feedback from invasive plant species. These studies evaluated aspects related to plants such as above-ground and underground biomass, but nothing related to the SMC; and (iii) studies where external factors were manipulated; e.g., addition of nitrogen and phosphorus, different levels of disturbance.

The *effect size* (*E*) was calculated for zones invaded by established invasive plants vs. places without invasive plants using the Hedge distance (d), which estimates the effect as the proportional change in the response variables that results from invasion by plants, and indicates the magnitude and sign of the effect size. For each case, we extracted average values, standard deviation and sample size of invaded and non-invaded zones. The algorithm was:

1 $$d = \frac{{\left( {\overline{X}^{i} - \overline{X}^{ni} } \right)}}{S}J$$

$\overline{X}^{i}$ is the mean value of SMC traits with the invasive plant and $\overline{X}^{ni}$ is the mean value of SMC variables without invasive plants; $S$ is the pooled standard deviation of response variables; J is a correction factor that weights for the sample size (N) of treatments [16, 40, 47]. Positive *d* values indicate that response variables are higher in invaded areas than in non-invaded areas, while negative values indicate that response variables are higher in the absence of the invasive plant. Zero values indicates no effect at all. Total effect is the average effect across all case studies.

For exploring, if the *d* estimate follows a normal distribution, we drew a normal quantile plot, a graphical method where the standardized *effect size* for each case is plotted against its normal quantile values [50].

We conducted heterogeneity tests using a random-effects model for two reasons. First of all, it enabled the effect and magnitude of invasive plants on the microbial community to be determined (objective one), and also enabled the effect of allelopathic substances on SMC to be determined between (a) climate zones (temperate regions versus tropical/subtropical zones), (b) ecosystem type (forest versus non-forest), and (c) growth habit (invasive woody versus invasive herbaceous) (objective two). We then calculated the total heterogeneity (*Q*_*t*_) which assessed whether the effect sizes in all cases were homogeneous. A significant value indicates that the variance among different effect sizes is greater than that expected by chance [40, 47]. We also calculated *Q*_*b*_ which describes the variation in effect sizes that can be ascribed to differences between categories (response variables). Finally, we applied *Q*_*w*_ which measures the statistical error of the data [40].

### Publication bias

In order to discern whether there was apublication bias, we used Spearman’s correlation test, which correlates the standardized effect size (*d*) with sample size (n). A significant positive correlation would suggest that there is a bias towards publishing cases that report large effect sizes [40].

All statistical and graphical analyses were carried out using MetaWin software [40].

## Supplementary Information

**Additional file 1:** Cases and studies that evaluated the impact of invasive plant species on soil microorganism communities. This table shows all references considered in meta-analysis. All data needed to calculate the effect size are included.

**Additional file 2:** Distribution pattern of each individual study. Graphical method where the standardized *effect siz*e of each individual study is plotted against its normal quantile value.

## Data Availability

All data generated or analysed during this study are included in this published article [and its Additional file 1].

## Abbreviations

SMC: Soil microorganism community
FLF: Free living fungi
EM: Ectomycorrhizal
AMF: Arbuscular mycorrhizal fungi
*E*: Effect size
*D*: Hedge distance
*Q*_*t*_: Total heterogeneity effect sizes
Q*w*: Heterogeneity within response variables
*Q*_*b*_: Effect sizes between categories
n: Sample size
