# Supplementary figures and images for: Meta-analysis of the impact of plant invasions on soil microbial communities
Source: BMC Ecol Evol. 2021 Sep 8;21:172. doi: 10.1186/s12862-021-01899-2 (PMC8425116; doi:10.1186/s12862-021-01899-2)

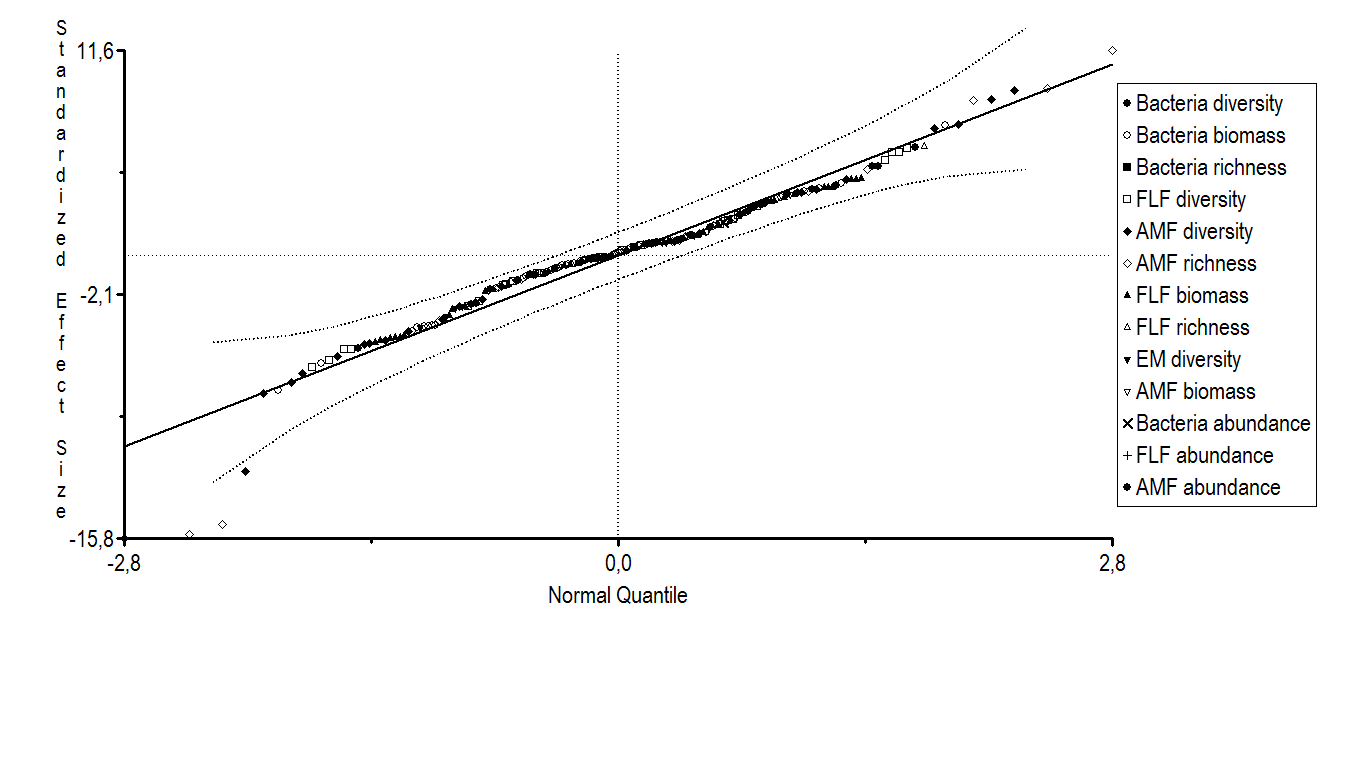

Supplement: Supplementary file 2 — Additional file 2: Distribution pattern of each individual study. Graphical method where the standardized effect size of each individual study is plotted against its normal quantile value. [file 12862_2021_1899_MOESM2_ESM.bmp]
